# Estimating Disability-Adjusted Life Years due to Tuberculosis in Korea through to the Year 2040

**DOI:** 10.3390/ijerph17165960

**Published:** 2020-08-17

**Authors:** Su Yeon Jang, Moon Jung Kim, Hyeon-Kyoung Cheong, In-Hwan Oh

**Affiliations:** Department of Preventive Medicine, School of Medicine, Kyung Hee University, 26 Kyungheedae-ro, Dongdaemun-gu, Seoul 02447, Korea; jcaecil@snu.ac.kr (S.Y.J.); plansby2020@naver.com (M.J.K.); chongmy99@hanmail.net (H.-K.C.)

**Keywords:** burden of disease, disability-adjusted life years, time series, tuberculosis, Korea

## Abstract

Despite efforts to reduce its incidence, tuberculosis continues to burden the rapidly aging Korean society. This study aimed to investigate the current trend of tuberculosis burden in Korea and its projections to 2040. We used National Health Insurance claims data to calculate the disability-adjusted life years due to tuberculosis in Korea. Disability-adjusted life years were measured by summing the years of life lost and the years lived with disability using an incidence-based approach. We modeled the incidence rates using a time-series model for the projection of disability-adjusted life years accrued from 2020 to 2040. The total disability-adjusted life years due to tuberculosis were 69, 64, 59, and 49 disability-adjusted life years/100,000 population in 2014, 2015, 2016, and 2017, respectively. In both sexes, disability-adjusted life years were the highest in those aged ≥80 years. Projected disability-adjusted life years showed a descending trend from 38 disability-adjusted life years/100,000 in 2020, to 14 disability-adjusted life years/100,000 in 2040. Conversely, the projected disability-adjusted life years increased among females aged ≥80 years. Although the tuberculosis burden in Korea is decreasing, a high burden remains among the elderly. Therefore, interventions targeting those vulnerable are required.

## 1. Introduction

Despite longstanding efforts to control the disease, tuberculosis (TB) is still one of the most burdensome diseases globally. It remains one of the top 10 causes of death from a single disease (drug-susceptible TB) worldwide and is the second-most frequent communicable-disease cause of death, following the lower respiratory disease group [1]. The United Nations (UN) set Sustainable Development Goal (SDG) Target 3.3 to end the TB epidemic by the year 2030, and the World Health Organization (WHO) reaffirmed this target with the End TB Strategy to reduce TB deaths by 95% and new cases of TB by 90% by 2035 [2,3].

Since 2013, Korea has been implementing its 5-year national strategic plan to end TB, known as the National TB Control Plan [4]. With continuous TB management, incident cases in Korea first dropped below 20,000 in 2017 [5]. However, as a rapidly aging society, Korea still faces a high burden of TB due to the disease prevalence among the elderly. Statistics Korea estimates that the proportion of the population aged 65 years and older will increase to 46.5% in the next 50 years [6]. The impact of population aging on the burden of TB in Korea is reflected by new notified cases, as the proportion of newly diagnosed patients aged 65 or older increased from 45.5% in 2018 to 47.1% the following year [7].

Disability-adjusted life years (DALYs) are often used to estimate the burden of disease because this measure includes information on both mortality and morbidity; DALYs are the sum of the years of life lost (YLLs) and the years lived with disability (YLDs), which in turn represent the burden from death and illness, respectively [8]. According to estimates from the Global Burden of Disease (GBD) studies, TB contributed to 6.5% of the total DALYs due to communicable diseases in all ages, and this increased to 17.0% of DALYs in the population aged 65 years or older in 2017 [9]. In the Korean Burden of Disease studies, TB resulted in the biggest number of DALYs among communicable diseases in Korea, with approximately 98.5 DALYs/100,000 population [10]. Furthermore, the projected burden of disease in Korea shows that DALYs due to HIV/AIDS and TB are expected to increase in both males and females by 2030 when compared with that in 2015 [11].

As TB places a heavy burden on the aging Korean society, it is important to investigate the trends in TB burden over time, especially in age- and sex-specific groups. However, studies to investigate the burden of a single disease have mostly considered non-communicable diseases, with little research on communicable diseases available [12,13,14,15].

In Korea, all residents within the nation’s territory are eligible for universal health coverage through National Health Insurance (NHI) and the Medical Aid Program. These programs provide valid epidemiological indicators of the entire Korean population via health insurance claim data [16]. From the NHI claim data, it is possible to calculate the national burden of disease.

Therefore, this study aimed to use nationwide NHI claim data from the period 2006–2017 to determine the current TB burden and trends in Korea and to estimate the projected burden until the year 2040. In this study, detailed estimates of the YLLs, YLDs, and DALYs due to TB from 2014 to 2017, as well as projected estimates to the year 2040, were calculated by age and sex to show trends across groups.

## 2. Materials and Methods

### 2.1. Study Design and Data Sources

The data were collected from the NHI claims of the Korean population. We included participants who were diagnosed with TB-related International Classification of Diseases-10 (ICD-10) codes (A15–A19, P37.0, U84.3, U88.0, U88.1) and were dispensed at least one of the selected anti-TB medications following diagnosis from 2014 to 2017 (Appendix A; Table A1) [17]. Included participants were HIV/AIDS-negative patients who were newly diagnosed with active tuberculosis in the study period. Any individuals diagnosed with TB in previous years, HIV/AIDS positive, or latent TB-infected were excluded.

This study measured DALYs by summing the subcomponents of YLLs and YLDs using the incidence-based approach. The detailed method of DALY estimation is described elsewhere [18]. To assess YLLs, we used data on deaths caused by TB (A15–A19) and the life expectancy by year, sex, and age from the national life tables provided by Statistics Korea, enabling the estimation of Korean context-specific TB burden [19,20]. Specifically, the cause-of-death statistics and the national complete life table were used to calculate TB-specific mortality and the life expectancy in each 1-year age interval group. For the calculation of YLDs, TB duration and the age of onset were first estimated using the DisMod II program, which automatically computes the duration and age of TB onset when inputting incidence, mortality, and fatality rates into the program (Appendix B; Table A2 and Table A3). The incidence rate was calculated using NHI claims data; the mortality and fatality rates were determined by accessing death data. A disability weight of 0.333 was used for all three sequelae included (i.e., drug-susceptible TB, multidrug-resistant TB without extensive drug resistance, and extensively drug-resistant TB), as determined by the Global Burden of Disease study in 2013 [21]. For the YLLs, YLDs, and DALYs/100,000 population, we used the mid-year population by year, sex, and age, obtained from Statistics Korea [22].

### 2.2. Projection Methods for Disability-Adjusted Life Years

We used time-series modeling to estimate the DALYs. We modeled the incidence rates using an exponential smoothing state space model with trigonometric regressors to model multiple seasonalities, Box–Cox transformation, autoregressive moving average (ARMA) errors, trend, and seasonal components (TBATS model) [23]. The model and its compositional parameters are shown in Appendix C. TBATS uses a combination of Fourier terms with an exponential smoothing state space model and a Box–Cox transformation to evaluate multiple forecasting techniques. In the model, the ARMA filter had components of p = 0 and q = 0, while the seasonal periods were m1 = 1. The model with the lowest Akaike information criterion score was selected as optimal. We performed the sensitivity analysis by applying the moving block bootstrap technique suggested by Künsch [24] and Liu and Singh [25], through which the regeneration of auto-correlated time-series data is possible, to duplicate our results. The result from the moving block bootstrap simulation was adopted at the value 1. Time-series models were applied by using the forecast package in R Version 8.10, which uses R statistical software Version 3.6.1 (R Foundation for Statistical Computing, Vienna, Austria).

To predict future incidence and mortality rates, we used an incidence rate based on 2006–2017 NHI claim data and a mortality rate derived from the deaths classified by the A15–A19 TB codes in Statistics Korea from 1983 to 2018. The future population and life table of Korea were obtained from Statistics Korea [6,26]. Age of onset and the duration of TB in each year between 2020 and 2040 were computed using the DisMod II program by inputting the projected future incidence, mortality, and fatality rates. We assumed that the disability weight would stay at the same level determined for the years 2014–2017, as it did not show a change in its trend in those years.

### 2.3. Ethics Statement

This study was approved by Kyung Hee University’s Institutional Review Board (IRB No. KHSIRB-19-167(EA)). The requirement for informed consent was waived because we used de-identified public data from the NHIS database.

## 3. Results

### 3.1. Years of Life Lost, Years Living with Disability, and Disability-Adjusted Life Years due to Tuberculosis

**Figure 1:**
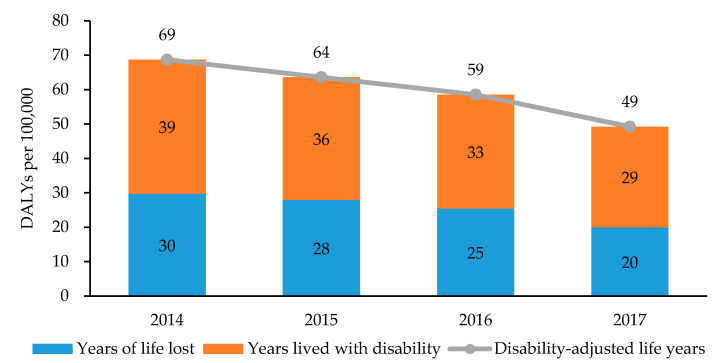
Years of life lost, years lived with disability, and disability-adjusted life years due to tuberculosis in Korea, 2014–2017.

Figure 1 shows the results of estimated YLLs, YLDs, and DALYs per 100,000 population due to TB from 2014 to 2017 in Korea. From 2014 to 2017, YLLs due to TB in Korea were 30, 28, 25, and 20 per 100,000 population, respectively, showing a descending trend; YLDs due to TB were 39, 36, 33, and 29 per 100,000 population, respectively; and the total DALYs per 100,000 population due to TB were 69, 64, 59, and 49, respectively.

From 2014 to 2017, YLLs due to TB were the highest in those aged 80 years and older, followed by those aged 70–79 years (Table 1). In 2017, TB-induced YLLs in the age group 80 years and older were 212 per 100,000 population in males and 125 per 100,000 population in females; among the 70–79 year age group, YLLs due to TB in 2017 were 109 per 100,000 population in males and 38 per 100,000 population in females.

The YLDs due to TB in Korea between 2014 and 2017 were the highest in those aged 80 years or older in males (Table 2). Among the females, the YLDs due to TB were the highest in the 20–29 year age group from 2014 to 2015 and highest in the 80 years or older age group from 2016 to 2017.

For each year between 2014 and 2017, DALYs attributable to TB in Korea were the highest in those aged 80 or above, followed by the 70–79 group in both sexes (Table 3). In 2017, DALYs due to TB in those aged 80 or above were 279 per 100,000 among males and 166 per 100,000 among females; also, TB-induced DALYs in the 70–79 age group were 164 per 100,000 population in males and 76 per 100,000 population in females.

### 3.2. Projected New Cases and Deaths due to Tuberculosis

**Figure 2:**
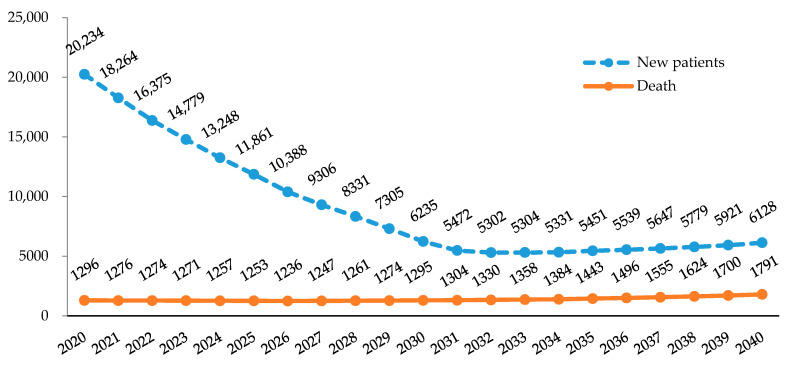
Projected number of new cases of tuberculosis and deaths caused by tuberculosis in Korea, 2020–2040.

Figure 2 shows the projected number of new TB patients and deaths caused by TB in Korea from 2020 to 2040, calculated by time-series modeling. It can be observed that the total number of cases is expected to decrease from 20,234 in 2020 to the inflection point of 5302 in 2032, before increasing slightly to 6128 by the year 2040. The number of deaths caused by TB between the years 2020 and 2040 shows a decreasing trend until the year 2026, from 1296 to 1236, before increasing again to 1791 in 2040.

**Figure 3:**
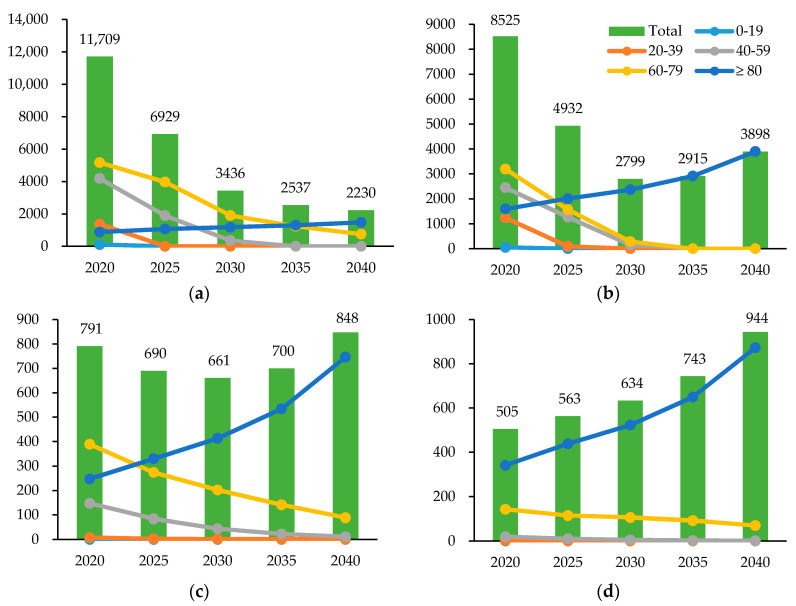
Projected number of new tuberculosis cases and deaths caused by tuberculosis in Korea by sex and age group, 2020–2040. (**a**) The projected number of new tuberculosis cases in males, by age group. (**b**) The projected number of new tuberculosis cases in females, by age group. (**c**) The projected number of deaths caused by tuberculosis in males, by age group. (**d**) The projected number of deaths caused by tuberculosis in females, by age group.

When estimated age- and sex-specifically, new cases of TB are expected to show a descending trend in total among the male population. In the female population, projected TB cases decrease from 8525 in 2020 to 2799 in 2030 and increase again to 3898 in 2040, due to the large growth rate in those aged 80 years and older (Figure 3a,b). The number of deaths due to TB shows a descending trend in all age groups below 80, while the age group 80 years or above showed a continuously increasing trend in both sexes (Figure 3c,d).

### 3.3. Projected Years of Life Lost, Years Living with Disability, and Disability-Adjusted Life Years due to Tuberculosis

**Figure 4:**
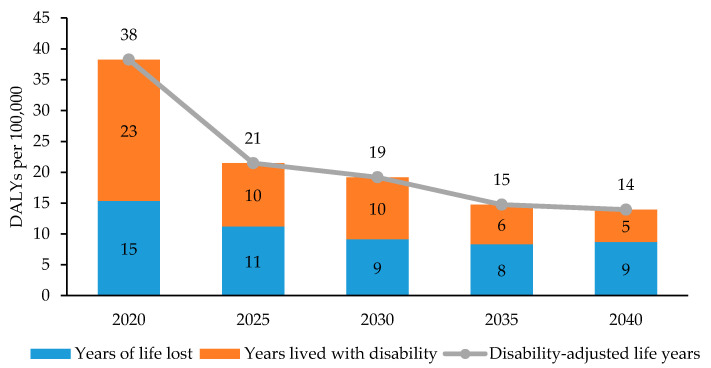
Projected years of life lost, years living with disability, and disability-adjusted life years due to tuberculosis in Korea, 2020–2040.

Figure 4 shows the results of projected YLLs, YLDs, and DALYs per 100,000 population due to TB from 2020 to 2040 in Korea. The number of DALYs per 100,000 due to TB is projected to be 38 in 2020, which will decrease to 14 in the year 2040. Both YLLs and YLDs are expected to show a continuous decrease. YLLs tend to account for an increasing proportion of DALYs due to TB than YLDs from 2020 to 2040.

**Figure 5:**
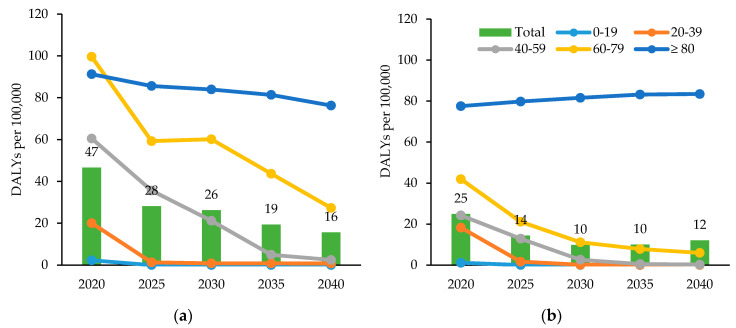
Projected disability-adjusted life years due to tuberculosis in Korea by sex and age group, 2020–2040. (**a**) The projected disability-adjusted life years due to tuberculosis in males, by age group. (**b**) The projected disability-adjusted life years due to tuberculosis in females, by age group.

Figure 5 illustrates DALYs per 100,000 population due to TB in sex- and age-specific groups, projected from the years 2020–2040. For every year from 2020 to 2040, the age group with the highest TB-incurred DALYs is the group aged 80 years and older, regardless of sex. In the male population, the projected DALYs per 100,000 population due to TB decrease from 47 per 100,000 in 2020 to 16 per 100,000 in 2040. In the female population, projected DALYs decreased in all age groups up to those aged 70–79 years, while those aged 80 years and older are expected to show an increasing trend during this period. DALYs due to TB in the entire female population are projected to be 25 per 100,000 in 2020, before decreasing to 10 per 100,000 in 2030 and increasing again to 12 per 100,000 in 2040.

## 4. Discussion

This study estimated the current trends and future projections of the TB burden in Korea through to the year 2040 and examined the burden in each age- and sex-specific group. According to the results, DALYs due to TB in Korea decreased over the study period from 2014 to 2017, with 69, 64, 59, and 49 DALYs/100,000 population, respectively. In 2017 in particular, TB-incurred DALYs by age and sex were the highest in males aged 80 or above (279 DALYs/100,000). Furthermore, the projected DALYs due to TB showed a descending trend over time until they reached 14 per 100,000 in 2040. The Korean Burden of Disease team previously assessed the burden of TB as 121 DALYs/100,000 population in 2012 and 98.5 DALYs/100,000 in 2015 [10,27]. Our findings that the overall TB burden in Korea showed a descending trend are consistent with previous studies.

In the GBD studies, TB-related burden in Korea from 2014 to 2017 was estimated to be 111, 107, 104, and 105 DALYs/100,000 population, respectively [28]. This is a considerably big amount of burden, as the TB-induced DALYs becomes more than twice than that of the current study in 2017. The difference is mostly from the YLLs due to TB for every year between 2014 and 2017, which are estimated to be much lower in our findings (30, 28, 25, and 20 per 100,000 population, respectively) than the GBD results (97, 93, 91, and 93 per 100,000, respectively); in contrast, YLDs from 2014 to 2017 in this study (39, 36, 33, and 29 per 100,000 population, respectively) are slightly higher than that of the GBD studies (14 per 100,000 in all four years, respectively) [28]. These discrepancies can be explained by the use of Korean-specific datasets, including the national complete life tables, cause of death statistics, and the health insurance claims data. Especially, this study used the life expectancy of the Korean population provided by Statistics Korea, which is higher than the standardized life expectancy used in the GBD studies [20,28]. Although the use of standard life expectancies may contribute to avoid the inequalities in life years between different regions globally and enable the comparison between countries, it may have overestimated the mortality-based burden of TB.

Overall, the burden of TB in Korea showed a decreasing trend and was expected to continue to decline over the next 20 years. Through the End TB Strategy, the WHO has developed targets to reduce 95% of deaths due to TB and 90% of the TB incidence globally by the year 2035 [3]. In accordance with the global target, the Korean government aims to reduce the national TB incidence to 40 per 100,000 population by the year 2022, and to end TB (i.e., incidence <10 per 100,000 population) by the year 2035 [4]. Due to continuous efforts to attain these internal and external goals, Korea has partially achieved its aim to reduce TB incidence: the total notified TB cases decreased from 89 per 100,000 population in 2013 to 77 per 100,000 population in 2016 [5]. Results from this study reflect the improved epidemiological indicator of fewer reported TB patients, demonstrating a reduced TB burden in Korea.

However, despite the declining trend in the overall burden of TB, Korea is still facing a challenge as the country with the highest TB incidence (66 per 100,000 population) among the 35 OECD (Organization for Economic Cooperation and Development) member nations [29]. Although notified TB cases have declined in recent years, Korea failed to meet the yearly goals of the First Comprehensive Plan for TB Management. The target number of total notified TB cases for 2016 was 72 per 100,000, while the actual number was 79 per 100,000; the target TB treatment success rate was 90% by 2016, but the actual rate achieved was 83.3%; the target number of TB-related deaths was 3.6 deaths per 100,000 for 2016, but the actual number was 5.2 deaths per 100,000 [4,5]. In this study, elderly females aged 80 years or older are expected to show an increase in TB-induced DALYs due to the increasing number of cases and deaths in the group. Furthermore, according to the estimates using the TBATS model, the predicted number of new cases of TB is expected to increase after 2032 and the number of TB-induced deaths after 2026, due to the increasing trends in the elderly population aged 80 years or above. Thus, continuous efforts are needed to reduce the burden of TB in Korea and achieve the worldwide goal to end TB by 2035.

We also assessed DALYs due to TB in Korea by sex and age. According to the study results, TB-incurred DALYs were higher in males than in females and in the older population compared to the younger population. In 2017, TB-incurred DALYs were the highest in the male group aged 80 years or above (279 DALYs/100,000). Similarly, previously published epidemiological indicators of TB showed a high burden of TB among the male and older populations [3]. Globally, previous findings on the gender disparities among the TB patients in Taiwan have determined that male-gender TB patients were more likely to be in the older age and to have comorbidities, which result in worse clinical outcomes compared to the female population [30]. Therefore, our findings suggest that targeted interventions in those vulnerable to TB, such as male and elderly populations, can be implemented to reduce the TB burden. For example, the Second Comprehensive Plan for TB Management includes a plan to implement a TB screening program for the elderly aged 65 or above; the plan involves utilizing laboratory vehicles to administer a mobile screening test (e.g., X-ray, sputum test) among the elderly population in regions with high TB incidence [4].

Previously, few studies have estimated the burden of TB globally. A study from China in 2017 reported that the DALYs attributable to TB were higher in males than in females and that the highest burden was found in the population aged 70 years or older, showing a similar result to our findings [31]. Similarities in Chinese and Korean trends in TB burden might be due to the relatively low incidence of HIV-positive TB in both countries compared with many countries with a high HIV prevalence, with 1.2 cases per 100,000 in China and 0.63 per 100,000 population in Korea [29]. In contrast to our findings, in Nigeria, the TB burden among HIV-negative individuals was the highest in those aged 15–49 years [32]. According to the GBD studies, DALYs due to the drug-susceptible TB among HIV/AIDS positives were the highest in males aged 30–39 globally [9].

Our study has several limitations. First, this study estimated the number of deaths due to TB in Korea as 1816 in 2017, which is considerably smaller than the 2500 deaths estimated by the WHO database [33]. TB-induced deaths in this study might have been underestimated due to the unspecified causes of deaths categorized under the garbage code, which were not included in the estimation of total TB deaths. In addition, due to the low HIV incidence and relatively insignificant comorbidity between TB and HIV in Korea compared with that in other countries, this study did not separately estimate the incidence of TB in those with or without HIV. Furthermore, the forecasting of deaths and new cases of TB followed the time-series modeling method, which cannot take unpredictable external changes that may occur in the future into account.

## 5. Conclusions

Despite the limitations, the study benefits from investigating current trends and future projections of TB-attributable DALYs from 2014 to 2040 by using the health insurance claim data of the entire Korean population. To our knowledge, this is the first study to investigate current and future trends in DALYs due to TB in Korea. Our findings suggested a generally decreasing trend in the burden of TB, though this may increase in the female elderly group in the future. Additionally, a high TB burden was found in male and elderly populations, reflecting the need for targeted policy implementation in vulnerable populations to end TB in Korea.

## Figures and Tables

**Table 1:** Years of life lost due to tuberculosis per 100,000 population in Korea by sex and age, 2014–2017.

Age Group	Male				Female			
	2014	2015	2016	2017	2014	2015	2016	2017
0–9	0	0	0	0	0	0	0	0
10–19	1	0	0	0	1	0	0	0
20–29	5	3	3	5	2	5	5	1
30–39	15	14	11	7	9	9	4	2
40–49	42	39	33	29	7	7	9	2
50–59	71	68	54	45	12	15	10	6
60–69	78	80	67	51	26	14	14	12
70–79	183	138	132	109	66	62	56	38
≥80	322	296	303	212	153	143	150	125
Total	43	40	35	29	17	16	16	11

**Table 2:** Years lived with disability due to tuberculosis per 100,000 population in Korea by sex and age, 2014–2017.

Age Group	Male				Female			
	2014	2015	2016	2017	2014	2015	2016	2017
0–9	5	3	4	1	5	6	1	1
10–19	17	16	27	11	14	24	10	8
20–29	53	44	41	29	56	53	41	35
30–39	46	40	36	32	44	38	34	30
40–49	49	44	41	35	34	28	26	26
50–59	56	52	51	47	32	28	25	25
60–69	56	53	53	49	33	29	27	27
70–79	65	59	58	55	48	45	42	38
≥80	68	63	63	67	46	44	44	42
Total	44	39	39	33	34	32	27	25

**Table 3:** Disability-adjusted life years due to tuberculosis per 100,000 population in Korea by sex and age, 2014–2017.

Age Group	Male				Female			
	2014	2015	2016	2017	2014	2015	2016	2017
0–9	5	3	4	1	5	6	1	1
10–19	18	16	27	11	16	24	10	8
20–29	57	47	44	34	58	58	46	36
30–39	61	54	47	39	53	46	38	32
40–49	91	83	74	64	41	35	34	28
50–59	127	120	105	92	44	42	35	31
60–69	134	134	121	100	59	42	41	38
70–79	248	197	190	164	114	107	98	76
≥80	390	359	366	279	199	187	194	166
Total	86	79	75	63	51	48	42	36

## Appendix A

**Table A1:** List of the anti-tuberculosis drugs selected for this study.

Classifications ^1^	Drugs (Abbreviations)
Single-agent products	
Group 1: First-line oral agents	• Isoniazid (H)
	• Rifampicin (R)
	• Ethambutol (E)
	• Pyrazinamide (Z)
	• Rifabutin (Rfb)
Group 2: Injectable agents	• Kanamycin (Km)
	• Amikacin (Amk)
	• Capreomycin (Cm)
	• Streptomycin (S)
Group 3: Fluoroquinolones	• Ciprofloxacin (Cfx)
	• Levofloxacin (Lfx)
	• Moxifloxacin (Mfx)
	• Ofloxacin (Ofx)
Group 4: Oral bacteriostatic second-line agents	• Prothionamide (Pto)
	• Cycloserine (Cs)
	• P-aminosalicyclic acid (PAS)
Group 5: Agents with an unclear role in the treatment of drug resistant-TB	• Linezolid (Lzd)
	• Clarithromycin (Clr)
	• Bedaquiline fumarate (Bdq)
	• Delamanid (Dlm)
	• Meropenem (Mpm)
Multiple-agent products	
Fixed-dose combination	• Isoniazid + Rifampicin (HR)
	• Isoniazid + Rifampicin + Ethambutol (HRE)
	• Isoniazid + Rifampicin + Ethambutol + Pyrazinamide (HRZE)
	• Isoniazid + Rifampicin + Pyrazinamide (HRZ)

^1^ Anti-tuberculosis drugs were classified according to the WHO guidelines [17].

## Appendix B

**Table A2:** Duration of tuberculosis in Korea in years by sex and age, 2014–2017.

Age Group	Male				Female			
	2014	2015	2016	2017	2014	2015	2016	2017
0–9	2.0	2.0	4.7	2.0	2.0	3.3	2.0	2.0
10–19	2.0	2.0	4.4	2.0	2.0	4.3	2.0	2.0
20–29	2.0	2.0	2.3	2.0	2.0	2.3	2.0	2.0
30–39	1.9	1.9	2.0	2.0	2.0	2.0	2.0	2.0
40–49	1.9	1.9	1.9	1.9	2.0	2.0	2.0	2.0
50–59	1.8	1.8	1.8	1.9	1.9	1.9	1.9	2.0
60–69	1.7	1.7	1.7	1.8	1.8	1.9	1.9	1.9
70–79	1.4	1.4	1.4	1.5	1.6	1.6	1.6	1.7
≥80	1.1	1.1	1.1	1.2	1.3	1.3	1.3	1.3

**Table A3:** Age of onset in Korea by sex and age, 2014–2017.

Age Group	Male				Female			
	2014	2015	2016	2017	2014	2015	2016	2017
0–9	5.2	5.6	6.1	6.7	5.1	5.4	6.1	6.4
10–19	16.0	16.0	16.2	16.2	16.1	16.2	16.4	16.4
20–29	25.2	25.1	25.1	25.2	25.3	25.2	25.2	25.2
30–39	35.1	35.2	35.2	35.4	34.9	34.9	35.1	35.2
40–49	45.2	45.3	45.4	45.5	44.9	45.0	45.1	45.2
50–59	55.0	55.1	55.2	55.3	55.0	55.1	55.2	55.2
60–69	64.9	64.9	64.9	64.8	65.1	65.1	65.1	65.0
70–79	74.8	74.9	75.0	75.1	75.1	75.2	75.4	75.4
≥80	84.5	84.5	84.5	84.5	85.3	85.4	85.4	85.5

## Appendix C

The TBATS model was calculated using the following equations:

Model:

(A1) $${y_{t}}^{\left(\lambda \right)}{=\;l}_{t-1}+\overset{T}{\underset{i=1}{\sum }}s_{{t-m}_{i}}^{\left(i\right)}{+\;d}_{t}$$

(A2) $$l_{t}{=\;l}_{t-1}+\phi b_{t-1}{+\;\alpha d}_{t}$$

(A3) $$b_{t}=\;\phi b_{t-1}{+\;\beta d}_{t}$$

(A4) $$d_{t}=\overset{p}{\underset{i=1}{\sum }}\varphi_{i}d_{t-i}+\overset{q}{\underset{i=1}{\sum }}\theta_{i}e_{t-i}{+\;e}_{t}$$

where

${y_{t}}^{\left(\lambda \right)}$: Time series at moment t (Box–Cox transformed),
${s_{t}}^{\left(i\right)}$: i-th seasonal component.

(A5) $${s_{t}}^{\left(i\right)}\;=\overset{\left(k_{j}\right)}{\underset{j=1}{\sum }}s_{j,\;t}^{\left(i\right)}$$

(A6) $$s_{j,\;t}^{\left(i\right)}{=\;s}_{j,\;t-1}^{\left(i\right)}\cos\left(\omega_{i}\right){\;+\;s}_{j,\;t-1}^{*\;(i)}{\sin(\omega }_{i}{)\;+\;\gamma }_{1}^{\left(i\right)}d_{t}$$

(A7) $$s_{j,\;t}^{\left(i\right)}{=\;-s}_{j,\;t-1}^{\left(i\right)}\sin\left(\omega_{i}\right){\;+\;s}_{j,\;t-1}^{*\;\left(i\right)}\cos\left(\omega_{i}\right){\;+\;\gamma }_{2}^{\left(i\right)}d_{t}$$

(A8) $$\omega_{i}{\;=\;2\pi j/m}_{i}$$

l_t_: Local level,
b_t_: Trend with damping,
d_t_: ARMA (p, q) process from residuals,
e_t_: Gaussian white noise.

Model parameters:

T: Amount of seasonality,
m_i_: Length of the i-th seasonal period,
k_i_: Number of harmonics for the i-th seasonal period,
λ: Box–Cox transformation,
α, β: Smoothing,
ϕ: Trend damping,
φ_i_, θ_i_: ARMA (p, q) coefficients,
$\gamma_{1}^{\left(i\right)}{,\;\gamma }_{2}^{\left(i\right)}$: Seasonal smoothing (two for each period).
